# Middle East respiratory syndrome coronavirus specific antibodies in naturally exposed Israeli llamas, alpacas and camels

**DOI:** 10.1016/j.onehlt.2018.05.002

**Published:** 2018-05-04

**Authors:** Dan David, Ditza Rotenberg, Evgeny Khinich, Oran Erster, Svetlana Bardenstein, Michael van Straten, Nisreen M.A. Okba, Stalin V. Raj, Bart L. Haagmans, Marcelo Miculitzki, Irit Davidson

**Affiliations:** a Kimron Veterinary Institute, Bet Dagan, Israel; b Hachaklait, Veterinary Services, Caesarea, Israel; c Department of Viroscience, Erasmus Medical Centre, Rotterdam, The Netherlands; d Beer Sheva District Director of Veterinary Services, Israel

**Keywords:** MERS coronavirus, Antibodies, Israel, Dromedary camels, Llamas, Alpacas

## Abstract

Thus far, no human MERS-CoV infections have been reported from Israel. Evidence for the circulation of MERS-CoV in dromedaries has been reported from almost all the countries of the Middle East, except Israel. Therefore, we aimed to analyze MERS-CoV infection in Israeli camelids, sampled between 2012 and 2017. A total of 411 camels, 102 alpacas and 19 llamas' sera were tested for the presence of antibodies to MERS-CoV. Our findings indicate a lower MERS-CoV seropositivity among Israeli dromedaries than in the surrounding countries, and for the first time naturally infected llamas were identified. In addition, nasal swabs of 661 camels, alpacas and lamas, obtained from January 2015 to December 2017, were tested for the presence of MERS-CoV RNA. All nasal swabs were negative, indicating no evidence for MERS-CoV active circulation in these camelids during that time period.

## 1 Introduction

Middle East respiratory syndrome coronavirus (MERS-CoV), a lineage 2C-betacoronavirus, was first identified in 2012 [1]. Serologic surveys identified >90% MERS-CoV–specific antibody seroprevalence in adult dromedary camels (*Camelus dromedarius*) in many countries in the Middle East and Africa. Moreover MERS-CoV viral RNA was detected in the nasal swabs of dromedaries in Qatar, Oman, Saudi Arabia, Egypt and United Arab Emirates (UAE) [2].

Confirmed MERS human cases in Iran, Jordan, Kuwait, Lebanon, Oman, Saudi Arabia, Qatar, UAE and Yemen, were epidemiologically linked to camels, indicating camels as a potential source of human infections [3]. Alpacas (*Vicugna pacos*) and llamas (*Lama glama*) are also susceptible to experimental MERS-CoV infections [[4], [5], [6]] but only alpacas were found to be naturally infected until now.

Presently, the highest MERS-CoV prevalence in humans has been documented in Saudi Arabia, as reported by the World Health Organization, whereas no cases have been reported from Israel despite the occurrence human-camel contact.

Approximately 155,000 Bedouins, traditionally a semi-nomadic pastoralist population, inhabit the Negev Desert and breed camels, sheep and goats. The estimated dromedary camel population in Israel is 3000–5000. In addition, the largest herd of alpacas and llamas, outside South America, is in the Negev. Since the virus is circulating in the surrounding countries, we aimed to assess the presence of MERS-CoV specific antibodies and viral RNA among camelids in Israel.

## 2 Material and methods

### 2.1 Samples

**Fig. 1:**
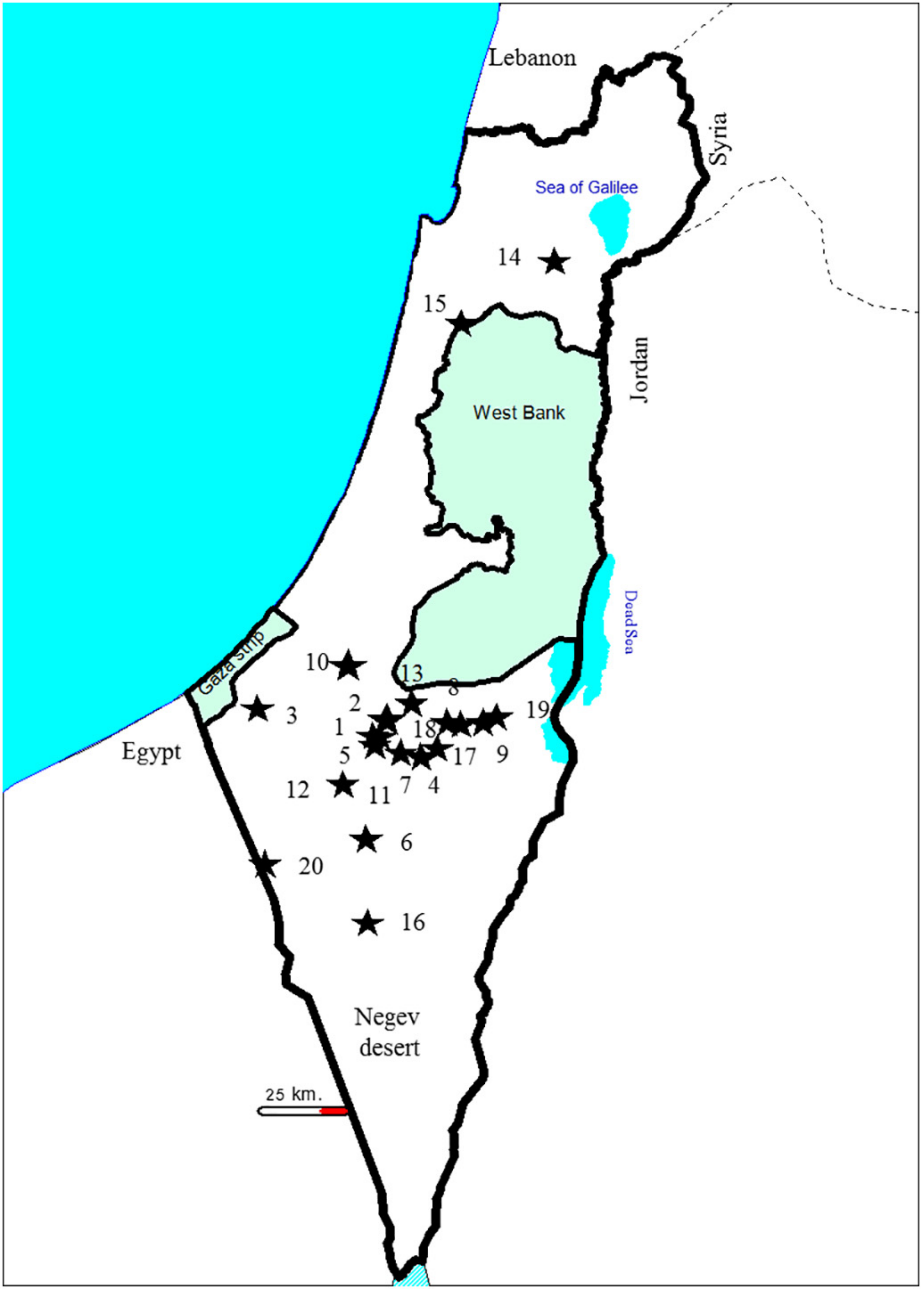
Locations of camelids sampled for MERS CoV monitoring.

A total of 411 blood samples from dromedary camels, 102 from alpacas and 19 from llamas were collected during 2012–2017 (Table 1, Fig. 1). The sampled camels were from 20 farms, 18 located in the Negev, one in central Israel and one in the north (Fig. 1). The camels included 37 were males and 374 were females, and their ages ranged from 1 month to 12 years. The alpacas and lamas sampled from one farm located in the Negev. The alpacas include 65 females and 37 males and their ages ranged from 6 month to 15 years. The lamas include 8 females and 11 males and their ages ranged from 2 years to 20 years.

**Table 1:** Total sera collected between 2012 and 2017, by location and species.

No.	Village	Year of Collection						Total
		2012	2013	2014	2015	2016	2017	
		Camels						
1	Mas'udin Al Azazme	4	5	–	11	16	15	51
2	Segev- Shalom	2	–	–	–		–	2
3	En Habesor	12	10	–	31	20	–	73
4	Aroer	9	–	–	–		–	9
5	Tarabin AS-Sani	4		6	39		19	68
6	Sede Boqer	7	2	–	–	1	–	10
7	Abu Qureinat	4	–	–	12		–	16
8	Abu Rubeia	9	–	–	3	22	27	61
9	Qabboa	–	3	–	–		9	12
10	Rahat	–	–	2	2	18	10	32
11	Azem	–	2	–	1	3		6
12	Revivim	–	–	–	14	14	–	28
13	Hura	–	–	–	1			1
14	Shibli	–	2	–	–		–	2
15	Arara	–	7	–	–			7
16	Mitzpe Ramon					2	–	2
17	Ksifea					1	24	25
18	Tel Sheva						1	1
19	Nokdim					3		3
20	Azuz					2		2
	Total camels	51	31	8	114	102	105	411

Alpaca								
	Mitzpe Ramon					102		
	Total					102		102

Llamas								
	Mitzpe Ramon					19		
	Total					19		19

A total of 540 nasal swabs from camels, 102 from alpacas and 19 from llama were collected between January 2015 and November 2017. Camel samples were collected from 486 females and 54 males, aged from 1 month to 19 years. The alpacas and lamas were from one farm in the Negev.

### 2.2 Virus neutralizing antibodies test (VNT)

The virus neutralizing antibodies test (VNT) is the gold standard assay for the serological diagnosis of the MERS-CoV infection. The camel, alpaca and llama sera were tested at a dilution of 1:20–1:2560 for the presence of neutralizing antibodies to MERS-CoV by the virus neutralization test (VNT) [7]. Briefly, sera were heat-inactivated by incubating for 30 min at 56 °C. Two-fold serial dilutions of sera were prepared in 96-well plates, starting at a 1:10 dilution. Sera reacting from a dilution of 1:20 and up were considered positive. Live MERS-CoV was diluted in Iscove's Modified Dulbecco's Medium (IMDM), supplemented with Penicillin, Streptomycin and 1% FBS, to a dilution of 2000 TCID_50_/ml. Subsequently, 50 μl virus suspensions was added to each well of plates and the plates were incubated at 37 °C for 1 h. Next, virus-serum mixtures were incubated on 96 well plates containing Vero cells for 1 h followed by washing with PBS and incubation with IMDM-1% FBS for 5 days, after which the cytopathic effect was scored.

### 2.3 ELISA

MERS-CoV antibodies were screened by ELISA (Euroimmun AG, Lubeck, Germany), according to the ELISA manufacturer's instructions. Briefly, diluted serum samples (1100) were incubated in ELISA plate wells, coated with MERS-CoV S1 antigen. Positive, negative and calibrator samples were included. Antibodies were detected by adding peroxidase-labeled rabbit anti–camel IgG. Results were reported as the optical density (OD) ratio, which was calculated as the OD value of the sample divided by the calibrator OD value. We used cutoff values recommended by the ELISA kit manufacturer: a ratio of <0.8 was considered negative, ≥0.8 to <1.1 was considered borderline, and ≥1.1 was considered positive [8]. Borderline reacting sera were not included in the comparison.

### 2.4 RNA extraction and detection by real-time RT-PCR

The swab specimens were suspended in 2 ml PBS, incubated for 1 h at room temperature and then clarified by centrifugation at 1000 rpm for 10 min. The supernatants were recovered for extraction and were stored at −80 °C until analysis. Total nucleic acid was extracted from 200 μl swab samples using Invisorb Spin virus RNA mini kit (STRATEC, molecular GmbH, Berlin, Germany), according to the manufacturer's instructions. Extracted RNA was tested for the presence of MERS-CoV RNA by real-time reverse transcription-quantitative polymerase chain reaction (qrt RT-PCR) hydrolysis probe assay using Bio Rad CFX 96 Real Time detection system (Bio Rad, Hercules, CA, USA). The primers and probe encompassed upstream the envelope gene (UpE) [9].

### 2.5 Statistical method

Serological data were entered into an Excel worksheet and analyzed using Excel functions. Results from Excel were verified using public access statistical software (https://www.medcalc.org/calc/diagnostic_test.php, http://vassarstats.net/kappa.html, http://vassarstats.net/prop1.html

## 3 Results

**Fig. 2:**
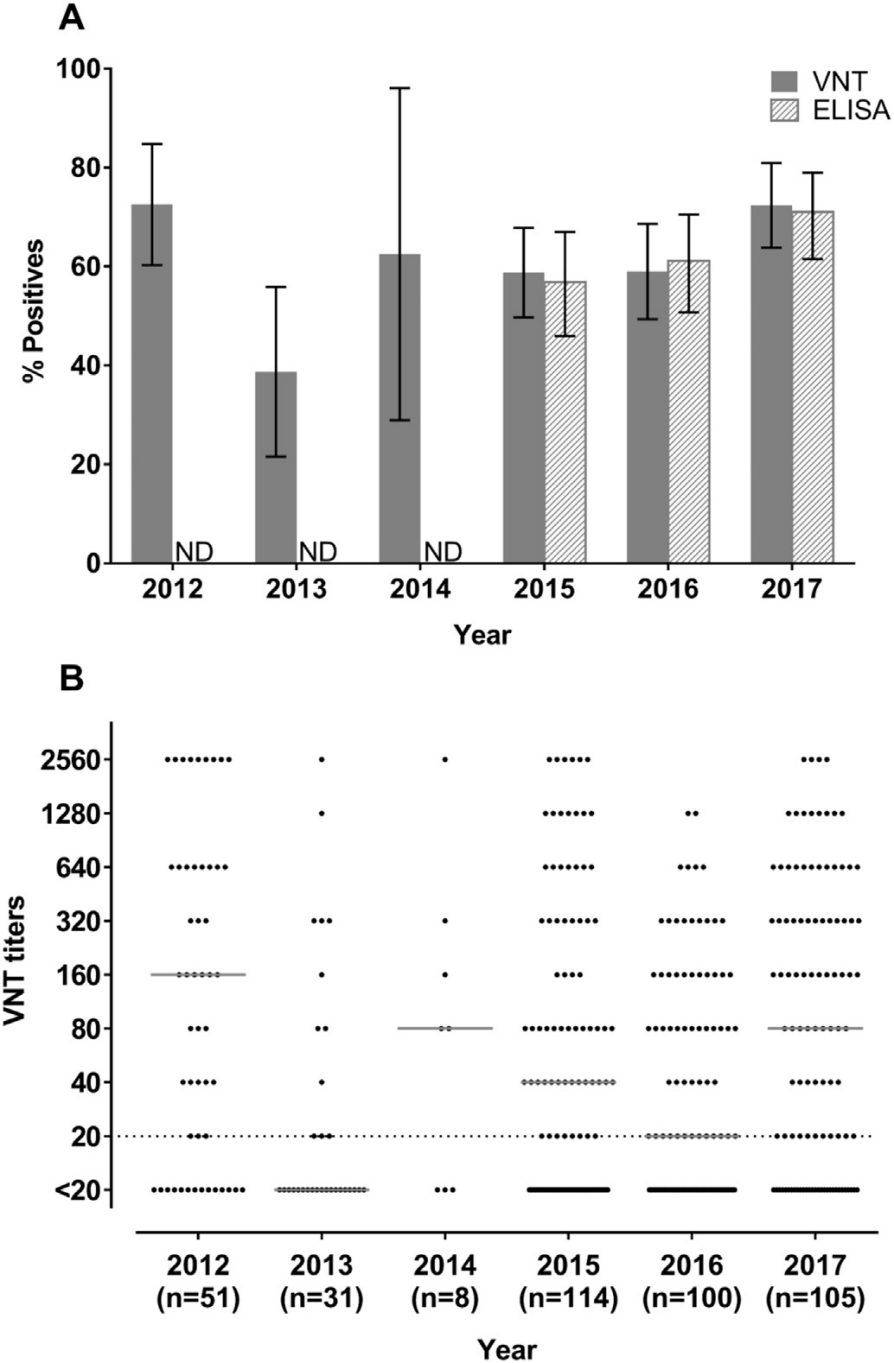
Detection of MERS-CoV antibodies in sera of dromedary camels, Israel 2012–2017. A) Percentage of MERS-CoV antibody positive by VNT and ELISA. Point estimate proportions (PEP) and confidence intervals (CI) are given per year. B) Serum neutralizing antibody titers by VNT. Red line indicated median; dotted black lines at 20 indicates cutoff for the VNT assay. (For interpretation of the references to colour in this figure legend, the reader is referred to the web version of this article.)

MERS-CoV specific antibodies in Israeli camels were analyzed using two serological assays, VNT and ELISA. Until the year 2014 sera were analyzed only by VNT, as being a gold standard, performed by the reference laboratory. With the expansion of screening activities in Israel, in subsequent years, sera were analyzed by ELISA also, as enabled by safety requirements, thus both assays were performed in parallel. Fig. 2 shows the neutralization titers and the percentage positives with confidence level (CI) detected by the two assays per year. While the VNT was performed on all sera collected from 2012 to 2017, ELISA was performed only on the sera collected between 2015 and 2017.

We initially tested 90 camel sera, obtained from 2012 to 2014, using VNT. Most camels were adults, 3 years and more, thus analysis of infection by age is not possible. MERS-CoV neutralizing antibodies for the year 2012 showed a seropositivity of 72.5% point estimate proportion (PEP) (CI: 60.3%, 84.8%) at a 95% confidence level (CL). The seropositivity seemed to drop to 38.7%, during 2013. Sera from 2015 to 2017 were analyzed by both ELISA and VNT (Fig. 2A, B). The seroprevalence of MERS-CoV antibodies obtained using ELISA did not significantly differ from those obtained using VNT, as both PEPs and Cls overlapped (Fig. 2). Most camel sera were collected from the Southern part of Israel, except of 2 farms (nos. 14 and 15) (Table 1, Fig. 1), from the Northern and Central Israel, respectively. Only one camel serum from farm no. 14 was positive.

The sensitivity and specificity of the two serological assays, VNT and ELISA, between 2015 and 2017 were determined on 271 camels sera that were tested in parallel by the two assays. Taking VNT as the gold-standard the sensitivity of the ELISA when compared to the VNT assay was 91.71% (CI: 86.70%, 95.29%) and the specificity was 93.33% (Cl: 86.05%, 97.51%).

We performed a kappa evaluation of the agreement between the ELISA and the VNT tests, receiving a value of 0.82 (CI: 0.75, 0.89; 95% CL). As defined by Cohen [10], 0.82 shows a perfect strength of agreement.

Overall for 2012 to 2017, 254 camels (61.8%) were VNT positive, with neutralizing titers from 20 to ≥2560 (Fig. 2A). In 184 camels (70.4%), low antibody titers, between 20 and 320, were measured, while 70 camels (29.6%) had high antibody titers, between 320 to ≥2560. No antibodies were detected (titer < 20) in 155 (37.8%) camels.

**Fig. 3:**
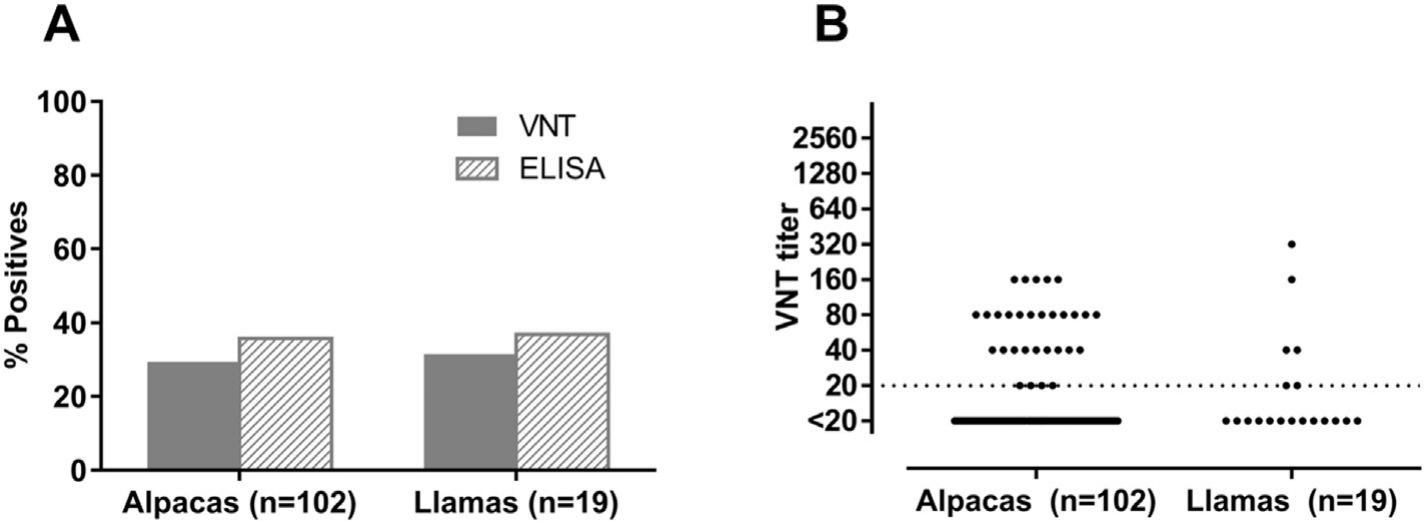
Detection of MERS-CoV antibodies in sera of alpacas and lamas by the VNT and ELISA. A) Percentage of MERS CoV antibody positive by VNT and ELISA per year. B) Serum neutralizing antibody titers by VNT. Dotted black line at 20 indicates cutoff for the VNT assay.

During 2016, blood was collected from 102 alpacas and 19 llamas and tested by ELISA and by VNT (Fig. 3A). Using ELISA the alpaca and llama sera showed a seroprevalence of 34.3 and 36.8%, respectively (35 and 7 positives). Using VNT, 30 alpacas and 6 llamas were positive, and PEPs were 29.4% (CI: 21.4%, 38.8%) and 31.5% (CI: 15.3%, 54.0%) respectively. The VNT titers were relatively low, ranging from 20 to 320 (Fig. 3B). The two camels sampled from the same farm as the alpacas and lamas were negative for MERS-CoV by both VNT and ELISA.

The nasal swabs of 540 camels, 102 alpacas and 19 llamas, collected between January 2015 and December 2017, were analyzed for the presence of MERS-CoV RNA. All nasal swabs were negative for the presence of MERS-CoV RNA, providing no evidence for active circulation, at least until 2017, of MERS-CoV in the Negev camels.

## 4 Discussion

We present the first serological and molecular survey for MERS–CoV infection among Israeli camelids, from 2012 to 2017. Camels showed an average MERS–CoV seroprevalence of 60.6% between 2012 and 2017 by VNT an average seroprevalence of 62.9% by ELISA between 2015 and 2017, without any detection of viral RNA in the nasal swabs of the 540 camels. The presence of virus-specific neutralizing antibodies suggests that Israeli camels were exposed to MERS-CoV in the past. The VNT assay was more sensitive than the ELISA, which had only 91.7% sensitivity when compared to VNT assay. That feature could be related to the fact that the ELISA coating antigen was the MERS-CoV spike S1 protein, whereas the VNT assay employed complete virions that could reveal antibodies against other viral proteins. Alternatively, whereas the ELISA detects only IgG, the VNT detects any isotype of antibodies which could be IgG, IgM or IgA. The VNT assay was also more specific than the ELISA, which had a calculated specificity of only 93.33% when compared to VNT. In addition, the higher strength of the VNT assay as compared to ELISA might be attributed by the fact that borderline sera by ELISA were not considered.

For the period from 2012 to 2017, the proportion of camel sera with high VNT titers (>320) in Israel was 27.3%, which is much lower than the proportion of sera with high VNT titers in the surrounding countries, such as Saudi Arabia, Qatar, UAE, Oman, Egypt, and Jordan, reaching to >90% [2, [11], [12], [13], [14], [15].]. Moreover, the proportion of sera with low VNT titers (<320) in Israel was about 70.4%, whereas in in the surrounding countries only about 10% of the sera had low VNT titers meaning that the MERS-CoV seroprevalence in Israel is lower than that in the surrounding countries. The differences in seropositivity by year in Israel might be related to the camels included in the random sample.

In contrast, low seroprevalence and probably no virus circulation were documented in the Canary Islands (14.3%) [14].

In the present study we showed for the first time the presence of MERS-CoV antibodies in naturally infected llamas by ELISA and VNT. In our study 29.4 and 31.5% of Israeli alpacas and llamas, respectively, were seropositive using the VNT assay. Using ELISA only 34.3 and 36.8% of alpacas and llamas, respectively were detected seropositive. The two camels co-housed with the llamas and alpacas were negative for antibodies and viral RNA using the two serological assays and PCR, respectively. Previously MERS–CoV neutralizing antibodies were detected in 15/15 alpacas in Qatar [16]. Similar to our findings, lower neutralizing antibody titers were documented in alpacas and experimentally infected llamas than in camels [5,16].

Using a MERS-CoV specific RT-PCR we detected no MERS-CoV genomic RNA in the nasal swabs of camels, alpacas and llamas during the period of 2015 to 2017 providing no evidence for active MERS-CoV circulation.

## 5 Conclusions

Altogether, the relatively low prevalence of antibodies to MERS-CoV, the low antibody titers, the absence of viral RNA in nasal swabs, and the absence of disease in humans indicate, limited MERS-CoV circulation in Israeli camelids in during the time of our survey.
